# A Qualitative Study on Barriers to Hospital at Home

**DOI:** 10.1093/geroni/igaf122.852

**Published:** 2025-12-31

**Authors:** Panpan Wang, Ying Qin, Mengting Liu, Changqing Sun

**Affiliations:** Zhengzhou University, Zhengzhou, Henan, China (People’s Republic); Zhengzhou University, Zhengzhou, Henan, China (People’s Republic); Zhengzhou University, Zhengzhou, Henan, China (People’s Republic); Zhengzhou University, Zhengzhou, Henan, China (People’s Republic)

## Abstract

As the process of population aging accelerates and the pressure on family caregiving continues to increase in China, building a comprehensive service system that can both meet patients’ home healthcare needs and effectively alleviate the burden on family caregivers has become an urgent task. Hospital at Home, as an important model of integrated healthcare and eldercare, not only provide continuous home medical care for those with mobility impairments or chronic diseases but also help alleviate the strain on healthcare resources to some extent. However, despite the gradual promotion of this service model across various regions, its implementation is still constrained by multiple factors. Following the concept mapping methodology, this study systematically explores the main barriers to the promotion of Hospital at Home through in-depth interviews with the medical professionals of different community medical institution. The study first classifies the identified barrier factors and uses expert scoring methods to assess the priority and feasibility of addressing each factor. Next, through multidimensional scaling and hierarchical cluster analysis, a concept map is constructed to analyze the relationships between the various factors. Finally, based on this analysis, the study explores the integration mechanism of respite care, aiming to achieve a complementary and collaborative relationship between medical support and social services. The theoretical significance of this study lies in two aspects: on the one hand, it provides empirical evidence to improve the Hospital at Home system, and on the other, it offers theoretical guidance for promoting the implementation of respite care services in primary healthcare institutions.

